# Different Transcriptomic Responses to Thermal Stress in Heat-Tolerant and Heat-Sensitive Pacific Abalones Indicated by Cardiac Performance

**DOI:** 10.3389/fphys.2018.01895

**Published:** 2019-01-09

**Authors:** Nan Chen, Zekun Huang, Chengkuan Lu, Yawei Shen, Xuan Luo, Caihuan Ke, Weiwei You

**Affiliations:** ^1^State Key Laboratory of Marine Environmental Science, Xiamen University, Xiamen, China; ^2^College of the Environment and Ecology, Xiamen University, Xiamen, China; ^3^College of Ocean and Earth Sciences, Xiamen University, Xiamen, China; ^4^State-Province Joint Engineering Laboratory of Marine Bioproducts and Technology, College of Ocean and Earth Sciences, Xiamen University, Xiamen, China

**Keywords:** Pacific abalone, heat stress, cardiac performance, ABT, transcriptome

## Abstract

The Pacific abalone *Haliotis discus hannai* is one of the most economically important mollusks in China. Even though it has been farmed in southern China for almost 20 years, summer mortality remains the most challengeable problem for Pacific abalone aquaculture recently. Here, we determined the different heat tolerance ability for five selective lines of *H. discus hannai* by measuring the cardiac performance and Arrhenius breakpoint temperature (ABT). The Red line (RL) and Yangxia line (YL) were determined as the most heat-sensitive and most heat-tolerant line, respectively. Heart rates for RL were significantly lower than those of the YL at the same temperature (*p* < 0.05). The differentially expressed genes (DEGs), which were enriched in several pathways including cardiac muscle contraction, glutathione metabolism and oxidative phosphorylation, were identified between RL and YL at control temperature (20°C) and heat stress temperature (28.5°C, the ABT of the RL) by RNA-seq method. In the RL, 3370 DEGs were identified between the control and the heat-stress temperature, while only 1351 DEGs were identified in YL between these two temperature tests. Most of these DEGs were enriched in the pathways such as protein processing in endoplasmic reticulum, nucleotide binding and oligomerization domain (NOD) like receptor signaling, and ubiquitin mediated proteolysis. Notably, the most heat-tolerant line YL used an effective heat-protection strategy based on moderate transcriptional changes and regulation on the expression of key genes.

## Introduction

The Pacific abalone (*Haliotis discus hannai*) is one of the most economically important mollusk species in China. The Pacific abalone is endemic to the coastal areas of Eastern Asia, including Northern China, Korea, and Japan (Li et al., 2007; Li J. et al., 2012; Chen N. et al., 2017). The large-scale Pacific abalone farming began in the late 1980s to cope with the decreasing of wild resources and increasing market demand (Guo et al., 1999; Li et al., 2007). Originally, most abalone farms were situated in coastal areas within or close to the range of wild abalone. However, new varieties of the Pacific abalone had been successfully introduced to locations outside of the endemic range since 2000, including subtropical areas such as Fujian province (Deng et al., 2008). In 2016, China produced nearly 140,000 tons of abalone, and Fujian accounted for 80% of that total production (Guo and Zhao, 2017). However, the water temperature in Fujian is much higher than that in the natural habitat of the Pacific abalone, which put tremendous pressure on abalone. The warmer temperature not only reduce oxygen solubility but increase the mollusk’s metabolic rate (Hamburger et al., 1994; Hoegh-Guldberg and Bruno, 2010; Vaquer-Sunyer and Duarte, 2011), which may trigger a rising number of oxygen deficient. The immune response of abalone could also be affected by the elevated temperature associated with increased in its susceptibility to disease outbreak (Cheng et al., 2004; Dang et al., 2012). Especially in summer, these influences would be more serious and the mass mortality occurred frequently (Liang et al., 2014). Therefore, it is important to explore how the abalone respond to the heat stress and whether different strategies are taken to adapt to the high temperature.

How do organisms response to the environmental change is one of the basic question for environmental physiology. Recently, advances in biotechnology have allowed experiments aimed at assessing this question to become increasingly reductionist, by relating changes in environment to genome-scale phenomena (Evans, 2015). Transcriptomics has been one of the popular methods because it can be used to compare the gene expression of different tissues and development stages with various treatment, even without the need for a reference genome (Martin and Wang, 2011; Shiel et al., 2015). The different trait related transcriptome studies had been widely performed on abalone (Franchini et al., 2011; van der Merwe et al., 2011; Valenzuela-Munoz et al., 2013; Choi et al., 2015; Picone et al., 2015; Valenzuela-Miranda et al., 2015; Harney et al., 2016). However, previous work on heat tolerance in the abalone was limited. Transcriptome-wide scans of the red abalone (*H. rufescens*) from three environmentally distinct regions were analyzed, and some loci associated with the heat response were identified (Wit and Palumbi, 2013). In the green lip abalone (*H. laevigata*), transcriptome analyses indicated that the heat shock protein 70 (HSP 70) gene family was strongly associated with heat response (Shiel et al., 2015). The related genes and transposable elements that respond quickly and effectively to summer heat stress (20°C) have been identified in heat-resistant green lip abalone line (Shiel et al., 2017). In southern China, ambient water temperatures can be over 28°C in summer, which was much higher than those published abalone heat test on red and green lip abalones. It would be very interesting to conduct the transcirptome analyses for the Pacific abalone under such high temperature and analyze whether there are similar or different heat response mechanism on different abalone species.

Different environments may exert strong selection pressures on different populations, leading to local adaptations to particular conditions (Hereford, 2009; North et al., 2011; Wit and Palumbi, 2013). Typically, such natural selection should produce phenotypes sensitive or resistant to a particular environmental stressor. In ectothermic organisms, physiological performance is sensitive to ambient temperature variation and is closely linked with the organismal heat tolerance (Portner et al., 2006; Clark et al., 2008). The Arrhenius breakpoint temperature (ABT) of cardiac performance has been used as an indicator for heat tolerance in porcelain crabs (*Petrolisthes*) (Stillman and Somero, 1996), marine snail (*Tegula*) (Stenseng et al., 2005), limpets (*Cellana*) (Dong and Williams, 2011; Han et al., 2013), and scallops (Xing et al., 2016). The ABT also has been shown to be a credible, non-invasive indicator of heat tolerance in abalone (Chen et al., 2016; Alter et al., 2017).

The study’s objective was to identify differences in patterns of gene expression between heat-sensitive and heat-tolerant abalones when subjected to heat stress on subtropical area. We first aimed to assess heat tolerance with ABT measurements in various selective lines of *H. discus hannai*. Based on this assessment, we were able to identify heat-sensitive and heat-tolerant abalone lines, and to conduct transcriptome analyses. We also aimed to identify genes or pathways associated with heat response in abalones by comparing gene expression patterns between sensitive and tolerant lines. The results might provide clues as to how abalone cope with life at high temperature, increase our understanding of the thermal response patterns of the Pacific abalone and highlighted potentially important genes or pathways for future study.

## Materials and Methods

### Experimental Animals

Five Pacific abalone selective lines were used in this research for the heat tolerance assessment: Yangxia line (YL), Dongshan line (DL), Red line (RL), Changdao line (CL) and Japanese line (JL). 50 individuals with the same size (4.5–6 cm) from each line were selected, labeled and cultured in a recirculating system for 7 days acclimation. Temperature, dissolved oxygen and salinity were kept at 20°C, 6 mg/L and 32, respectively. All abalones were fed once each day with fresh seaweed and all the residual food particles and fecal debris were removed 12 h before the start of the experiment.

### Cardiac Performance

Sixteen individuals of each line were randomly selected, and crystal dishes (diameter = 20.0 cm, height = 9.5 cm) were used for abalone to settle. Fresh seawater was added into the dishes, and the dissolved oxygen concentration was kept by delivering air from a compressor to each dish. To measure real-time body temperature, a fine thermometer was inserted between the foot and the crystal dish. The crystal dish was immersed in a water bath, and the temperature of the seawater in the dish was increased using the water bath on the rate of approximately 0.1°C min^-1^.

The non-invasive method was used for heart rate measurement (Depledge and Andersen, 1990; as modified by Chelazzi et al., 1999; Dong and Williams, 2011). To detect heart beats, an infrared sensor was glued to each shell above the heart (Krazy Glue, Westerville, OH, United States). The fluctuations of heart beat were amplified, filtered and recorded by an infrared signal amplifier (AMP03, Newshift, Leiria, Portugal) and Powerlab (8/35, ADInstruments, March-Hugstetten, Australia). All heart rate data were monitored and analyzed with LabChart v8.0. The ABT was defined as the temperature at which the heart rate decreased dramatically. To determine the ABT, we used regression analyses to generate the best fit lines on both side of a putative break point (Stillman and Somero, 1996). To construct Arrhenius plots, heart rates were transformed to the natural logarithm of beats min^-1^. Temperatures are shown as 1000/K (Kelvin temperatures). To test the significance of differences in ABT and maximum heart rate among lines, we used one-way analyses of variance (ANOVAs) in SPSS v17.0. We considered *p* < 0.05 statistically significant.

### Heat Stress Experiments

Based on mean ABT for each line, we could distinguish the heat-sensitive lines from the heat-tolerant lines. The ABT of the most sensitive line was selected as the thermal stressor. We randomly selected an additional 24 specimens, 12 from the most heat-tolerant line and 12 from the most heat-sensitive line. Half of all specimens (6 per line) were randomly assigned to the control group. All others were assigned to experimental group. For heat exposure, the abalones were transferred to the experimental tank from the acclimation condition (20°C) directly, in which the temperature was set as ABT of most sensitive line prior to the experiment. The heat stress lasted for 2 h and the control group was kept at 20°C at the same time. All the abalones were dissected at the end of experiments and gills were immediately frozen in liquid nitrogen and stored at -80°C until use.

### RNA Preparation and Sequencing

Total RNA was extracted from the gills of the 24 samples using Trizol reagent (Gibco BRL, United States). To check the purity and integrity of RNA, the Nanophotometer^®^ spectrophotometer (IMPLEN, Westlake Village, CA, United States) and RNA Nano 6000 Assay Kit of the Agilent Bioanalyzer 2100 system (Agilent Technologies, Santa Clara, CA, United States) were used. Based on these results, we selected three individuals with the best RNA quality of six samples from each of the four groups (sensitive line/tolerant line × heat stress/control) for transcriptomic analyses. Library preparation and sequencing were performed by Novogene (Beijing, China). We obtained an average of ∼52.8 million raw reads per specimen (maximum: 63.0 million raw reads; minimum: 44.8 million raw reads).

### *De novo* Transcriptome Assembly and Annotation

Raw reads were cleaned by removing ambiguous reads (those with > 10% ambiguous nucleotides) and low-quality reads (those consist of more than half bases whose quality scores < 10) to achieve clean reads. All clean data were assembled with Trinity r20140413p1 (Grabherr et al., 2011), with min_kmer_cov set to 2 and other parameters set to defaults. The longest transcript for each gene was considered to represent this gene and named unigene. Unigenes were used for all subsequent annotations.

To comprehensively annotate the assemblies, transcripts were compared to several public databases: Nt (NCBI non-redundant nucleotide sequences), Nr (NCBI non-redundant protein sequences), and Swiss-Prot using BLAST+ v2.2.28+ (e-value = 1e-5; Altschul et al., 1990); GO (Gene Ontology) with BLAST2GO (e-value = 1e-10; Götz et al., 2008); and KO (KEGG Orthologs) with KAAS r140224 (e-value = 1e-10; Mao et al., 2005; Moriya et al., 2007).

### Quantification and Differential Analysis of Gene Expression

The *de novo* assembled transcriptome was used as a reference for read mapping and gene expression profiling. To identify differential gene expression, the sequence reads for each specimen were mapped back to the assembled transcriptome using RSEM v1.2.1.5 (bowtie2 set mismatch = 0; Li and Dewey, 2011). The gene expression levels were based on FPKM values (expected number of Fragments Per Kilobase of transcript sequence per Millions base pairs sequenced). We compared differential gene expression between pairs of groups with DESeq v1.10.1 in R (Anders and Huber, 2012). The strict filter (*p*-value < 0.05 and |log2 (fold change)| > 1) was set as the threshold for significant differential expression. We performed four analyses to identify differential gene expression between the two lines or stress conditions: (I) the heat-sensitive line vs. the heat-tolerant line at the control temperature; (II) the heat-sensitive line vs. the heat-tolerant line at the heat stress temperature; (III) the heat-sensitive line at the control temperature vs. the heat stress temperature; and (IV) the heat-tolerant line at the control temperature vs. the heat stress temperature.

### Functional Annotation and Transcript Validation

Gene Ontology (GO) enrichment analysis of the differentially expressed genes (DEGs) was implemented by the GOseq v1.10.0 in R packages (Young et al., 2010). GOseq is based on Wallenius non-central hyper-geometric distribution, which adjusts for DEG gene length bias. We used KOBAS v2.0.12 (Mao et al., 2005) to test the statistically significant enrichment of DEGs in KEGG pathways.

The further validation for the RNA-seq results was conducted by real-time quantitative PCR (RT-PCR) with all the 24 individuals which were prepared in Section “Heat Stress Experiments.” The expression levels of 10 genes which selected from intriguing pathways were quantified. Based on the reference unigene sequences, we designed gene-specific primers with Primer Premier v5.0 (Supplementary Table S1). First-strand cDNA was synthesized with a PrimeScript RT reagent Kit (TaKaRa, Dalian, China), and qRT-PCR was performed with a ThermoDyNAmo Flash SYBR Green qPCR Kit (Thermo Scientific, United States) on an ABI 7500 Fast Real-Time PCR system (Applied Biosystems, United States), following the manufacturer’s instructions. Gene expression was normalized based on the expression of 18S and β-actin. Relative gene expression was then calculated using the 2^-ΔΔCT^ method.

## Results

### Cardiac Performance and ABT Measurement

**FIGURE 1:**
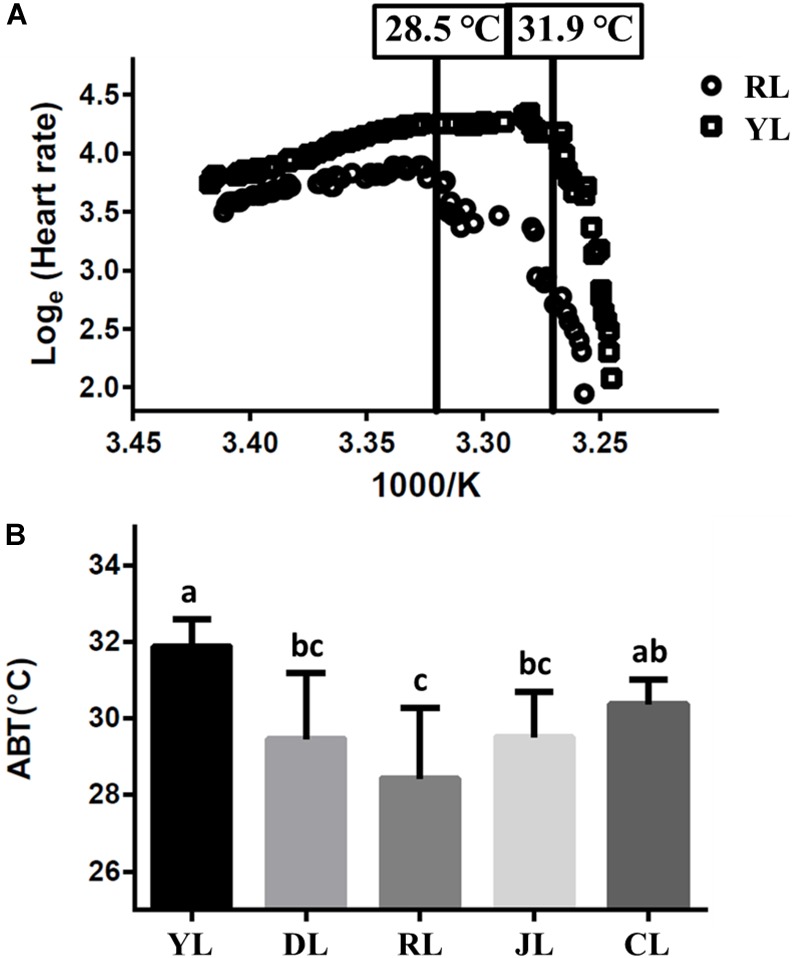
The Arrhenius breakpoint temperature (ABT) for different Pacific abalone lines. **(A)** The Arrhenius plots for YL and RL. **(B)** The ABTs for the five selective lines. Bars labeled with different letters are significantly different (*p* < 0.05).

The cardiac performance at different temperatures were shown by the Arrhenius plots (Figure 1A). The cardiac performance could be divided into two phases. Abalone heart rates initially increased slowly with rising water temperature. As water temperature continued to rise, incidents of arrhythmia were observed. Heart rates decreased abruptly as soon as the water temperature exceeded a critical value (the ABT). The ABTs for the YL, DL, RL, JL, and CL were 31.9 ± 0.7, 29.5 ± 1.7, 28.5 ± 1.8, 29.5 ± 1.2, and 30.4 ± 0.6°C, respectively (Figure 1B) using regression analysis. The ABT of the YL was significantly higher than that of the DL, RL, and JL. The ABT of the CL was also significantly higher than that of the RL. The YL was the most tolerant to heat stress, while the RL was most sensitive. Therefore, the individuals from YL and RL were selected for transcriptome analysis, and 28.5°C (RL’s ABT) was set as heat stress temperature. Heart capacity also differed between the RL and YL. Heart rates of RL specimens were lower than those of YL at the same temperature. The maximum of heart rates was also significantly different (Duncan’s *post hoc* analysis, *p* = 0.001; RL: 54.75 ± 6.90 beats min^-1^ and YL: 75.14 ± 7.29 beats min^-1^).

### *De novo* Assembly and RNA-seq Mapping

After filtering, a total of 43.2 to 60.3 million clean reads for each sample were obtained. The *Trinity* assembly generated 227,066 transcirpts and 161,135 unigenes, with the minimum length of 201 bp and the maximum length of 17 kb. Only 27.26% of all unigenes could be annotated in one or more databases. Approximately 70% of all reads were successfully mapped back to the reference transcriptome (68.12–72.33%) (Supplementary Table S2).

### Differentially Expressed Genes (DEGs)

**FIGURE 2:**
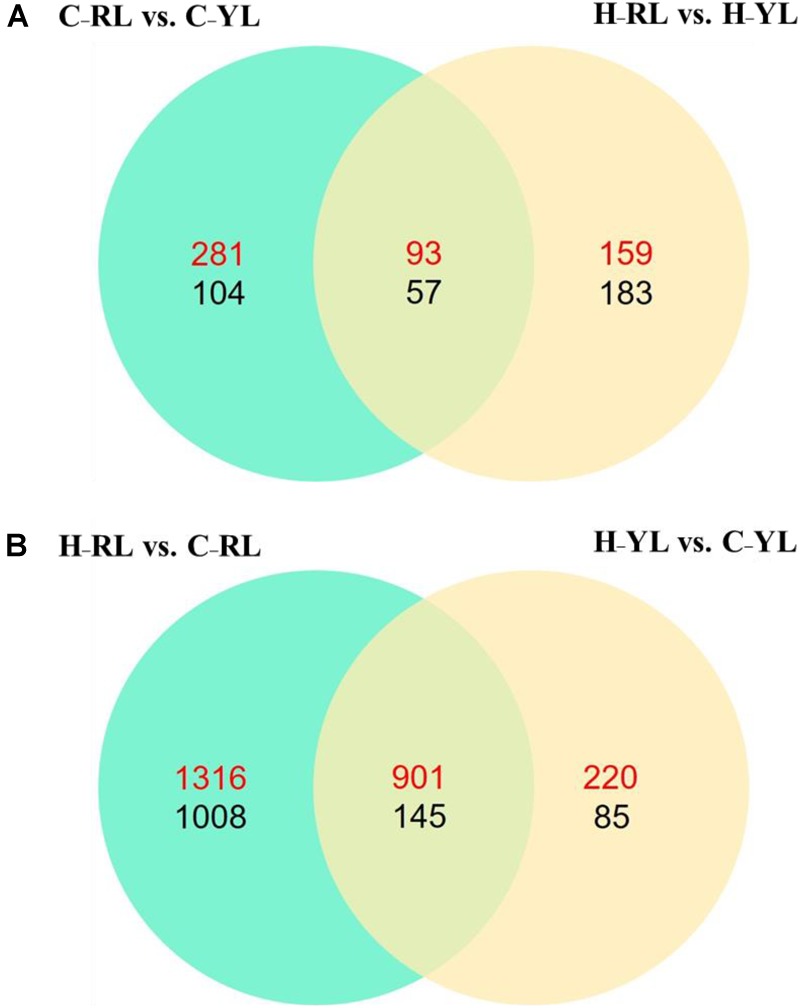
The Venn diagram of significant differently expressed genes. **(A)** Comparison between different lines. **(B)** Comparison between control temperature and heat stress temperature. C_RL: RL at control temperature; C_YL: YL at control temperature; H_RL: RL at heat stress temperature; H_YL: YL at heat stress temperature. Up-regulated genes were numbered in red and down-regulated genes were numbered in black.

We identified 535 DEGs between the heat-tolerant and heat-sensitive lines at the control temperature (20°C), and 492 DEGs at the heat stress condition. Of these DEGs, 150 were differentially expressed at both temperatures (Figure 2A). However, more genes were differentially expressed between the two temperatures as compared to those between the two lines. In the YL, 1351 DEGs were identified between the control and heat stress temperature: 1121 were up-regulated at the heat stress temperature as compared to the control, and 230 were down-regulated. Many more DEGs were identified in the RL. In the RL, 2217 DEGs were up-regulated at the heat stress temperature as compared to the control, and 1153 genes were down-regulated (Figure 2B). Of these DEGs, 1046 genes were differentially expressed in both lines.

### Gene Ontology (GO) Enrichment Analysis

**FIGURE 3:**
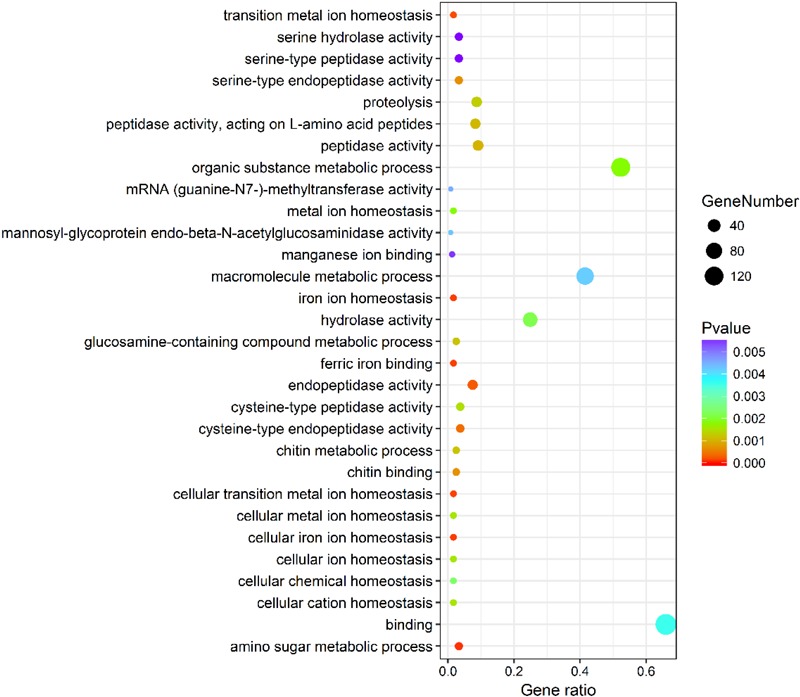
Top 30 GO terms enrichment statistics between RL and YL at control temperature.

**FIGURE 4:**
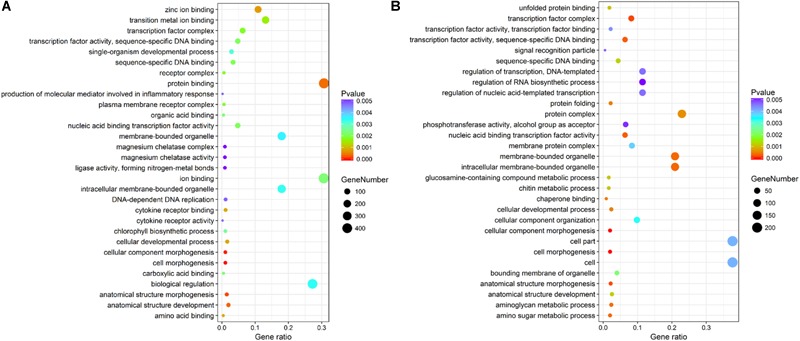
Top 30 GO terms enrichment statistics between control and heat stress temperature. **(A)** Terms enrichment in RL. **(B)** Terms enrichment in YL.

The genes differentially expressed between the RL and YL were enriched in several interesting GO terms, including organic substance metabolic process, macromolecule metabolic process, hydrolase activity, and binding (Figure 3). Between control and heat stress treatment, the DGEs were significantly enriched in protein binding, ion binding, biological regulation, and intracellular membrane-bounded organelle for RL (Figure 4A) and transcription factor complex, intracellular membrane-bounded organelle, protein complex for YL (Figure 4B). Of these, nearly twice as many genes were enriched for each term in the RL as compared to YL (Supplementary Table S3).

### KEGG Pathway Enrichment Analysis

**FIGURE 5:**
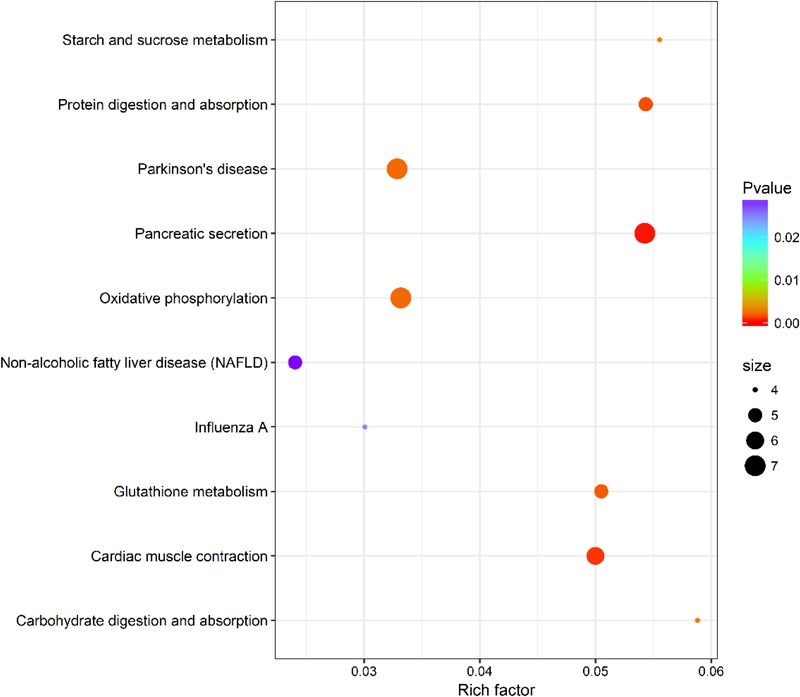
Top 10 pathways enrichment statistics between RL and YL at control temperature.

**FIGURE 6:**
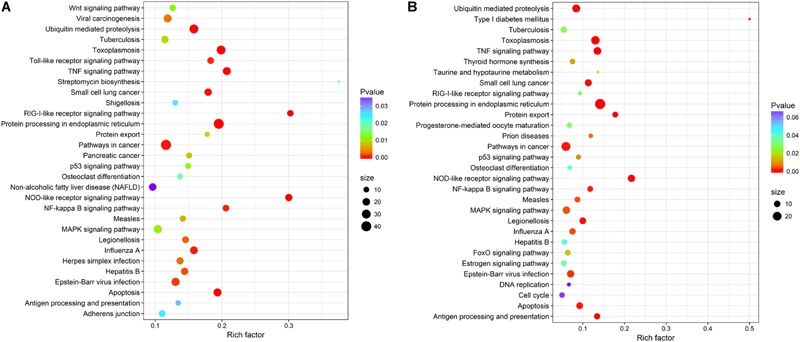
Top 30 pathways enrichment statistics between control and heat stress temperature. **(A)** Pathway enrichment in the RL. **(B)** Pathway enrichment in the YL.

Within KEGG pathway analysis, the results showed that the DEGs between the RL and YL were most enriched in the following five pathways: pancreatic secretion, cardiac muscle contraction, protein digestion and absorption, oxidative phosphorylation, and Parkinson’s disease (Figure 5). Between the control and heat stress temperature, DEGs were most significantly enriched in the protein processing in endoplasmic reticulum pathway: 40 RL and 29 YL DEGs were enriched in this pathway. Several other pathways were also significant up-regulated, including ubiquitin mediated proteolysis, apoptosis, nucleotide binding, and oligomerization domain (NOD-like) receptor signaling pathway and TNF signaling pathway (Figures 6A,B).

### Heat Shock Protein Family

**FIGURE 7:**
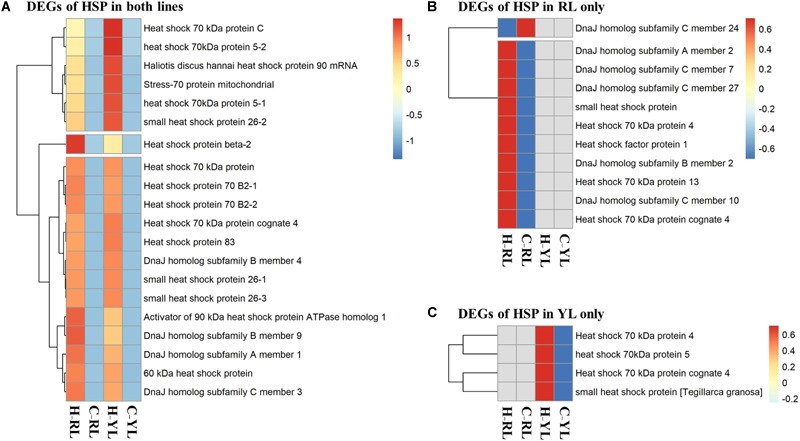
Heatmaps of differentially expressed heat shock protein (HSP) genes. **(A)** Genes differentially expressed in both lines. **(B)** Genes differentially expressed in the RL only. **(C)** Genes differentially expressed in the YL only.

Several genes in the HSP gene family were enriched in multiple pathways. Indeed, the expression levels of 35 HSP genes were significantly different between control and heat stressed specimens (Figure 7 and Table 1). Of these, 20 HSP genes were differentially expressed in both abalone lines, 11 were differentially expressed in the RL only, and 4 were differentially expressed in the YL only. The degree of up-regulation also differed among the differentially expressed HSP genes: from 1.6-fold (log_2_ Fold Change) (DnaJ homolog subfamily C member 7) to 11.9-fold (small heat shock protein 26) in RL and 2.9-fold (stress-70 protein, mitochondrial) to 11.4-fold (Heat shock 70 kDa protein) in YL.

**Table 1:** Differentially expressed heat shock protein family genes.

Gene name	Readcount			Readcount		
	H_RL	C_RL	Log_2_ fold change	H_YL	C_YL	Log_2_ fold change
**DEG in both lines**						
60 kDa heat shock protein	22940.4	1697.5	3.8	20808.9	1119.9	4.2
Activator of 90 kDa heat shock protein ATPase homolog 1	5238.0	55.9	6.6	3873.5	50.9	6.2
DnaJ homolog subfamily A member 1	14258.0	185.5	6.3	11714.6	111.0	6.7
DnaJ homolog subfamily B member 4	71733.8	474.1	7.2	76468.9	365.2	7.7
DnaJ homolog subfamily B member 9	442.4	19.8	4.5	318.0	16.1	4.3
DnaJ homolog subfamily C member 3	1816.7	101.8	4.2	1586.5	83.3	4.3
*Haliotis discus hannai* heat shock protein 90 mRNA	18359.5	290.8	6.0	29654.7	230.6	7.0
Heat shock 70 kDa protein	517199.6	157.6	11.7	512778.1	193.8	11.4
Heat shock 70 kDa protein C	40.2	3.1	3.7	91.9	2.7	5.1
Heat shock 70 kDa protein cognate 4	200721.2	6687.8	4.9	224124.7	4084.2	5.8
Heat shock 70 kDa protein 5-1	89771.3	3321.8	4.8	135757.5	2553.6	5.7
Heat shock 70 kDa protein 5-2	124.3	11.6	3.4	257.6	13.5	4.3
Heat shock protein 70 B2-1	1026410.4	601.0	10.7	953619.4	531.1	10.8
Heat shock protein 70 B2-2	103617.9	120.2	9.8	98169.6	108.6	9.8
Heat shock protein 83	351431.0	6616.4	5.7	389863.4	4789.3	6.3
Heat shock protein beta-2	266.7	4.7	5.8	137.1	2.5	5.8
Small heat shock protein 26-1	104467.4	28.1	11.9	109373.2	42.0	11.3
Small heat shock protein 26-2	81010.9	25.4	11.6	118746.7	46.2	11.3
Small heat shock protein 26-3	754714.9	417.6	10.8	795488.0	395.2	11.0
Stress-70 protein, mitochondrial	4156.8	1003.4	2.1	6168.8	854.8	2.9
**DEG in RL only**						
DnaJ homolog subfamily A member 2	5218.5	1508.9	1.8			
DnaJ homolog subfamily B member 2	308.6	95.0	1.7			
DnaJ homolog subfamily C member 10	23.3	2.8	3.0			
DnaJ homolog subfamily C member 24	25.0	120.8	-2.3			
DnaJ homolog subfamily C member 27	42.0	7.7	2.4			
DnaJ homolog subfamily C member 7	3339.3	1067.1	1.6			
Heat shock 70 kDa protein 13	32.4	5.6	2.5			
Heat shock 70 kDa protein 4	133179.5	549.7	7.9			
Heat shock 70 kDa protein cognate 4	80171.5	1847.4	5.4			
Heat shock factor protein 1	235.3	44.7	2.4			
Small heat shock protein	112213.5	112.6	10.0			
**DEG in YL only**						
Heat shock 70 kDa protein 4				152438.4	406.7	8.6
Heat shock 70 kDa protein cognate 4				75878.2	1199.4	6.0
Heat shock 70 kDa protein 5				28.2	0.9	4.9
Small heat shock protein [Tegillarca granosa]				131461.7	144.5	9.8

*RL, red line; YL, Yangxia line; C_RL, RL at control temperature; C_YL, YL at control temperature; H_RL, RL at heat stress temperature; H_YL, YL at heat stress temperature.*

### Ubiquitin-Mediated Proteolysis Pathway

**FIGURE 8:**
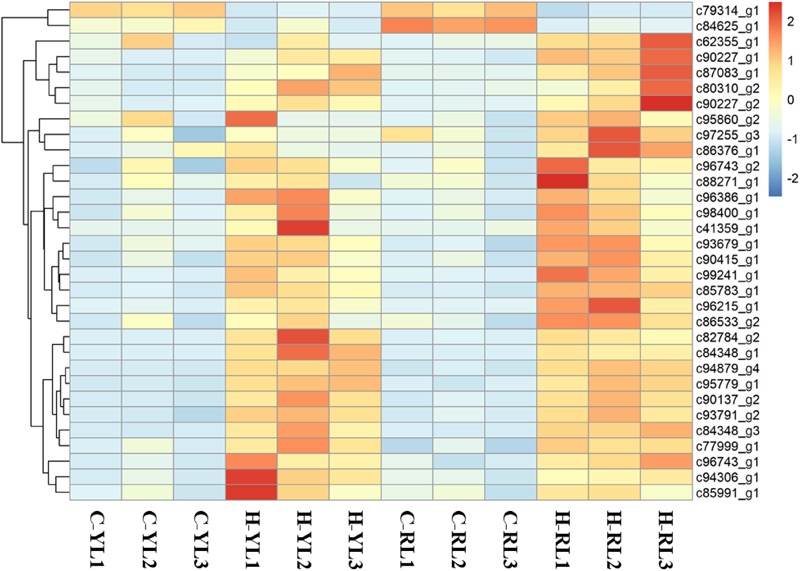
Heatmap of genes involved in the ubiquitin-mediated proteolysis pathway.

Ubiquitination serves as a versatile post-translational modification in all eukaryotic species. As shown in Figure 8, a total of 32 genes that involved in this pathway expressed differently. Of these, 16 genes and 30 genes were up regulated significantly in YL and RL, respectively.

### Antioxidant Genes

**FIGURE 9:**
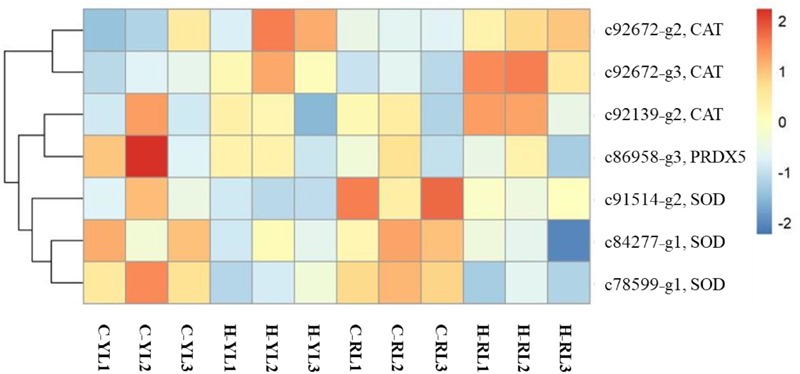
Heatmap of differentially expressed antioxidant-related genes. CAT, catalase; SOD, superoxide dismutase; PRDX5, peroxiredoxin 5.

Heat stress can result in oxidative stress as the formation of reactive oxygen species (ROS). There were several genes (including catalase, superoxide dismutase, peroxiredoxin 5) related to antioxidant system that annotated in this study (Figure 9) and only one catalase gene (c92672_g3) was up-regulated at heat stress temperature of both lines.

### Real-Time Quantitative PCR Validation

**FIGURE 10:**
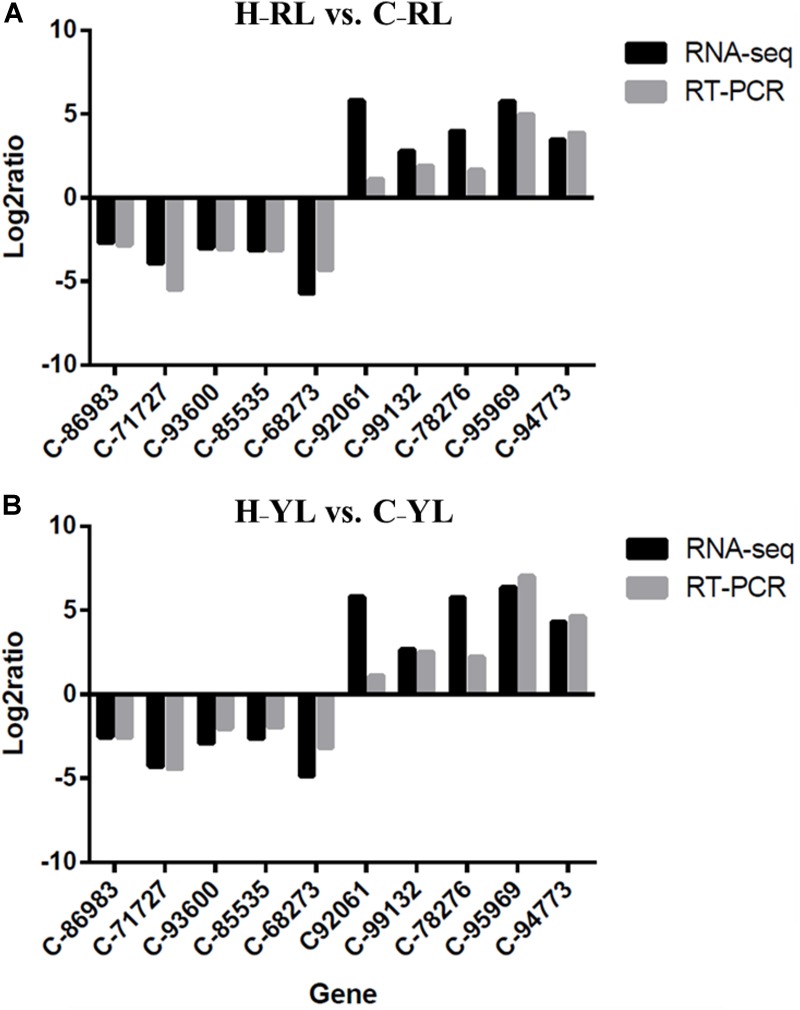
Comparison of qRT-PCR and RNA-seq results. **(A)** H_RL vs. C_RL; **(B)** H_YL vs. C_YL.

We used 10 genes that were differentially expressed between the control and heat stress temperatures for qRT-PCR validation. These DEGs included HSP90 (c_95969___g1), HSP70 (c_92061___g1) and cyclin B (c_86983_g1). The patterns of gene expression indicated by the qRT-PCR and RNA-seq analyses were similar (Figure 10).

## Discussion

### Cardiac Performance and Heat Tolerance Assessment

In ectothermic animals, temperature is the main variable which controls the rate of most metabolic processes (Morash and Alter, 2015). Increased temperatures can diminish oxygen solubility and change the metabolic rates (Hoegh-Guldberg and Bruno, 2010; Vaquer-Sunyer and Duarte, 2011). It thus becomes difficult for the organism to obtain sufficient oxygen to sustain aerobic scope, both with respect to providing metabolic fuel and to removing metabolic waste products at high temperature (Somero, 2012). Aerobic scope drops when the maximum metabolic rate fails to keep pace with the routine metabolic rate as temperature increases (Portner and Knust, 2007; Casselman et al., 2012). For many marine ectotherms, heat-stress tolerance is based on the ability to maintain normal aerobic scope in elevated water temperatures (Portner et al., 2006; Portner, 2010; Han et al., 2013). In gastropods, this capacity is largely dependent on heart function; the ABT of cardiac performance has thus been used as an effective indicator of heat tolerance in gastropods (Han et al., 2013; Chen et al., 2016).

We found that the five tested different Pacific abalone lines had different levels of heat tolerance. There were nearly 3.5°C difference between the most temperature-tolerant line (YL) and the most temperature-sensitive line (RL). At high water temperature, even a 1°C difference would affect abalone survival (Chen et al., 2016). The ABT for RL was 28.5 ± 1.8°C, which means RL abalone’s cardiac function is approaching the limit at this temperature. Thus, at 28.5°C, cardiac function in an RL individual is approaching the maximum, while the individuals of YL could maintain normal oxygen supply and cardiac function at the same temperature. The cardiac muscle contraction pathway was significantly down-regulated when compared control of RL to control of YL by RNA-seq results. Only six genes, including myosin light chain 4 (MYL 4) were involved in this pathway, *MYL 4* is particularly notable because this gene regulates several critical biological processes, including heart contraction force, muscle organ development, and cardiac muscle contraction. The MYL 4 protein is down-regulated in failing human hearts, as compared to normal human hearts (Li W. et al., 2012). To verify the difference in cardiac function, further study of the expression level of myosin family gene in the abalone heart is necessary.

### The Identified Different Expression Genes

Between the RL and the YL, 535 DEGs were identified at the control temperature, and 492 DEGs were identified at the heat-stress temperature. However, more genes were differentially expressed between the control and heat-stress temperatures: 1351 DEGs in YL and 3370 DEGs in RL. This increase transcriptome fluctuation in the more temperature-sensitive line has been shown in other organisms, including copepod (*Tigriopus californicus*; Schoville et al., 2012), coral (*Acropora hyacinthus*; Barshis et al., 2013), snail (*Chlorostoma funebralis*; Gleason and Burton, 2015), and redband trout (*Oncorhynchus mykiss gairdneri*; Chen Z. et al., 2017). Indeed, the increased heat-tolerance seems to be based on the moderate transcriptional changes under heat stress (Bita et al., 2011). Here, twice as many RL DEGs were enriched per GO term as compared to YL DEGs. However, it is possible that this discrepancy is due to the higher level of physiological stress experienced by the less thermally tolerant individuals. Thus, we speculated that the more effective protection strategy was not only based on fine-tuned but the more effective expression response in the heat-tolerant abalone line. In this research, we observed a higher degree change of gene expression in the YL, as compared to the RL in some key genes including HSP family.

The different physiological stress might also be indicated by the down-regulated pathways: cell cycle and DNA replication. In face of stress condition, cells often delayed the DNA replication and cell division in favor of cytoprotective functions (Jonas et al., 2013). More genes (Table 2) were down-regulated in sensitive line vs. tolerant line (9 vs. 5), which mean the cells of RL might be subjected to more severe suppression.

**Table 2:** Differentially expressed genes (DEGs) in cell cycle and DNA replication pathways.

Gene ID	Pathway	Log2 fold change		KO description
		H_RL vs. C_RL	H_YL vs. C_YL	
c68273_g1	Cell cycle	-5.7	-4.8	Cyclin A
c76770_g1	Cell cycle	-2.2	–	Polo-like kinase 1
c79314_g1	Cell cycle	-1.9	–	Anaphase-promoting complex subunit 12
c81134_g1	DNA replication	-2.2	–	Ribonuclease H2 subunit C
c86856_g2	DNA replication, cell cycle	-2.0	–	DNA replication licensing factor MCM6
c86983_g1	Cell cycle	-2.6	-2.5	Cyclin B
c88219_g1	DNA replication, cell cycle	–	-2.9	DNA replication licensing factor MCM3
c91413_g2	DNA replication	-1.8	–	Replication factor A2
c91496_g1	DNA replication, cell cycle	-1.8	–	DNA replication licensing factor MCM2
c93600_g1	DNA replication, cell cycle	-3.0	-2.9	DNA replication licensing factor MCM7
c94298_g1	DNA replication, cell cycle	-2.5	–	DNA replication licensing factor MCM5
c94298_g2	DNA replication, cell cycle	-3.1	-3.9	DNA replication licensing factor MCM5

*C_RL, RL at control temperature; C_YL, YL at control temperature; H_RL, RL at heat stress temperature; H_YL, YL at heat stress temperature.*

### HSP Family

Genes in the “Protein processing in endoplasmic reticulum” pathway, including many HSP genes, were significantly up-regulated. HSPs are highly conserved proteins that act as molecular chaperones to protect normal proteins from degeneration, catalyze the folding of normal proteins and the refolding of abnormal proteins, remove irreversibly damaged proteins, and help maintain cellular homeostasis (Snyder et al., 2001; Farcy et al., 2007; Liang et al., 2014). The molecular chaperones (HSP70, HSP90, and other HSPs) are one of the most varied gene families affected by heat stress in mollusks (Lang et al., 2009; Lockwood et al., 2010; Chapman et al., 2011). The rapid response of HSPs to heat stress is an important part of the molluscan protection mechanism (Zhao and Jones, 2012). There were fewer HSP DEGs in the YL (24) as compared to the RL (31), but the average fold change (log_2_ fold change) was higher in the YL (7.0) than in the RL (5.4). This indicated that the heat response strategy used by the YL might be more effective.

The HSP40 gene family is another important group, which primary function is to act as co-chaperones for HSP70 (Gleason and Burton, 2015). HSP40s increase the HSP70 ATP-hydrolysis rate and strengthen the HSP70 activity fields (Misselwitz et al., 1998; Genevaux et al., 2007; Mayer, 2013). In thermotolerant cells, this interaction might increase protein thermostability and accelerate heat-damage recovery (Gebauer et al., 1997; Terada et al., 1997; Gleason and Burton, 2015). The gastropod *C. funebralis* exhibited the opposite reaction to heat stress (Gleason and Burton, 2015): 66.7% of all annotated HSP70s showed a significantly higher fold change in heat-sensitive lines, as compared to heat-tolerant lines, while the expression levels of 60% of all HSP40s were higher in the heat-tolerant lines. Here, more than 70% of common HSP70 and HSP40 genes that differentially expressed in both lines showed a higher fold change in the YL, as compared to the RL. This might be because *C. funebralis* inhabits intertidal areas, where water temperature fluctuation is more drastic (Gleason and Burton, 2015) than in the subtidal habitat of the abalone. Some gene families, such as HSP60, are pre-adapted in heat-tolerant lines (Gleason and Burton, 2015), which might serve as a preparative defense against frequent heat stress events. In addition, significant up-regulation of HSP40 combined with reasonable up-regulation of HSP70 expression might reduce the need for the heat-tolerant lines to up-regulate HSP70 as drastically as the heat-sensitive populations do. For abalones, ambient water temperature does not fluctuate so frequently. Thus, the higher fold change of HSP gene family would be help to reduce the heat-damage more quickly in tolerant lines, which offered a more effective protection strategy.

### Ubiquitin, Antioxidant-Related Genes and “Preadaptation”

Genes involved in the ubiquitin-mediated proteolysis pathway were considered as key transcription factors that respond to abiotic stresses, acting to clear irreparably damaged proteins (Glickman and Ciechanover, 2002; Kültz, 2005; Lyzenga and Stone, 2012). The up-regulation of genes involved in proteolysis has been reported in *Mytilus trossulus* (Lockwood et al., 2010), *Saccharina japonica* (Liu et al., 2015), and *Pyropia haitanensis* (Wang et al., 2018b). The RL and YL showed similar induction of many genes involved in this pathway. However, the less DEGs occurred in YL may also indicate these two lines endured different physiological stress. More irreparably damaged protein may be accumulated in RL. Therefore, for RL, more genes may be need to participate in this response that was helpful to transfer more ubiquitin to damaged protein and target them for degradation in time.

Heat stress can induce the overproduction of ROS (Abele et al., 2002; Heise et al., 2003; Yang et al., 2010; Cui et al., 2011). ROS are toxic to the cell as they cause damage to macromolecules (Kültz, 2005). Then, mitigating oxidative stress by increasing synthesis of antioxidants can increase thermal tolerance (Dilly et al., 2012). It has been demonstrated that the genes of antioxidant defense system were up regulated in the study of blue mussel (*M. galloprovincialis* and *M. trossulus*) (Lockwood et al., 2010), coral (*Acropora hyacinthus*) (Barshis et al., 2013), snail (*C. funebralis*) (Gleason and Burton, 2015) and *P. haitanensis* (Wang et al., 2018a). However, few genes related to antioxidant system could be annotated in this study (Figure 9). This might be explained by the stress exposure time. Wang et al. (2018b) found that the expression of antioxidant related genes showed gradual up-regulation with sustained high temperature in *P. haitanensis*. The significant fold changes were observed at 2- or 6-day time points. The heat stress last a total of 5.5 h in study of snail (*C. funebralis*) (Gleason and Burton, 2015) and it was more than 4 h in blue mussel (*M. galloprovincialis* and *M. trossulus*) (Lockwood et al., 2010). While, it was just 2 h in this study. Therefore, more stress time points were necessary for future studying the response of antioxidant system.

Besides acute responsiveness, the thermally tolerant snail (*C. funebralis*) individuals appeared to also utilize the “preadaptation” to cope with heat stress (Gleason and Burton, 2015). Some genes, including HSP60, partly of superoxide dismutase (SOD), glutathione peroxidase (GPx), and ubiquitin showed higher constitutive expression in tolerant populations and less up-regulated following heat stress compared with sensitive ones. While, as to these gene or related families, the higher constitutive expression was not found in the tolerant population (YL). Therefore, the preadaptation could not be the abalone’s main strategy for coping with the heat stress. We speculated the better tolerance of YL may mostly rely on its precise coping strategy, which indicated by fine-tuning of transcirptome and stronger ability to regulate key genes.

## Conclusion

Our results suggest that the cardiac performance of thermally tolerant lines was less disrupted by high water temperatures stress. In addition, the heat-tolerant line employed a more effective strategy to cope with heat stress than the heat-sensitive line. This strategy included moderate changes in gene transcription and more effective regulation (such as more powerful to up-regulate the HSP family genes). Overall, our results provide insight into the different heat-response strategies employed by heat-tolerant and heat-sensitive abalone lines and these findings might provide some suggestions for further studies of heat-response mechanisms in mollusks. The key genes or pathways would be great indicator for our follow-up work. Combined these with the results with other studies, such as genome-wide association study (GWAS), would provide fundamental information for breeding of better heat-tolerant line.

## Ethics Statement

The methods were carried out in accordance with the approved guidelines by Laboratory Animal Management and Ethics Committee of Xiamen University. All experimental procedures involving abalones were performed according to the Regulations for the Administration of Affairs Concerning Experimental Animals (Xiamen University, China; revised in November 2014).

## Author Contributions

CK and WY conceived and designed the experiments. NC, CL, and YS performed the experiments. NC and ZH analyzed the data. XL offered reagents and experiment animals. NC, CK, and WY wrote the manuscript. All authors has reviewed the manuscript.

## Conflict of Interest Statement

The authors declare that the research was conducted in the absence of any commercial or financial relationships that could be construed as a potential conflict of interest.
