# Supplementary material for: Different Transcriptomic Responses to Thermal Stress in Heat-Tolerant and Heat-Sensitive Pacific Abalones Indicated by Cardiac Performance
Source: Front Physiol. 2019 Jan 9;9:1895. doi: 10.3389/fphys.2018.01895 (PMC6334008; doi:10.3389/fphys.2018.01895)
Supplement: Supplementary file 1 [file Table_1.pdf]

TABLE S1 **Details of primers used for real-time PCR (RT-PCR) analysis.**

**Gene ID**	**Forward Primer (5'-3')**	**Reverse Primer (5'-3')**
**c-86983-g1**	GTTCATCATCGCTTCCACTTACTG	TGTTCTTGCTCACTTGCTTCAC
**c-71727-g**	CGAACAAGATCACAGCGAGTA	GCGTGGAACATTGACGTAAGA
**c-93600-g1**	TATCAAGATGGCGACAAGA	CAGGTCAGGCTCATACTC
**c-95969-g1**	GAGATGGTAATGTGGCTGTTAAC	CTCTGGGAAGGTAGGTAGTGGT
**c-94773-g1**	CCATTCACGTCGATTTCAAAG	AAAGGTCATTCCTCGCAACA
**c-85535-g1**	GTGTTAGAAGAGGAGGTCGGG	TCCAGGCACTTTGGTCAGTAG
**c-68273-g1**	CGAAAGATAAGATAACCTCAGCTG	AGTAGGCATAACAATCACGAAA
**c-99132-g1**	TCTCGCTCCACGGTCCTCT	CCCAACCAGCAGTCTTGC
**c-78276-g1**	ACTCTTGCCACGCCCACT	CCGCGATTGCACTCTTATTC
**c-92061-g1**	AGCCGATTCTTTCCTATTACCT	TGAGCTTCCCTTGAAGTGAGT
**β -actin**	GGTATCCTCACCCTCAAGT	GGGTCATCTTTTCACGGTTG
**18S**	TTCCCAGTAAGCGTCAGTCATC	CGAGGGTCTCACTAAACCATTC

TABLE S2 **Number of clean reads and number of reads mapped to the *de novo* assembled transcriptome.**

Sample	Clean reads	Total mapped
C-YL1	52873890	37139760(70.24%)
C-YL2	48133616	32787754(68.12%)
C-YL3	43172594	29659766(68.70%)
H-YL1	48832832	35055752(71.79%)
H-YL2	53408264	37482876(70.18%)
H-YL3	60277900	43599676(72.33%)
C-RL1	50891928	35221528(69.21%)
C-RL2	46520868	32471742(69.80%)
C-RL3	46568540	32606398(70.02%)
H-RL1	60162314	43085218(71.61%)
H-RL2	49323292	35355100(71.68%)
H-RL3	49793126	35524582(71.34%)

TABLE S3 **Gene Ontology (GO) terms enriched at control and heat stress temperature for both lines**

Accession	Description	Term_type	DEG_item		Fold change (RL vs. YL)
			RL	YL	
GO:0000902	cell morphogenesis	biological_process	15	11	1.4
GO:0006355	regulation of transcription, DNA-templated	biological_process	138	63	2.2
GO:0006457	protein folding	biological_process	16	12	1.3
GO:0007049	cell cycle	biological_process	47	21	2.2
GO:0008643	carbohydrate transport	biological_process	13	6	2.2
GO:0009653	anatomical structure morphogenesis	biological_process	21	12	1.8
GO:0009889	regulation of biosynthetic process	biological_process	147	65	2.3
GO:0010468	regulation of gene expression	biological_process	144	64	2.3
GO:0010556	regulation of macromolecule biosynthetic process	biological_process	147	65	2.3
GO:0015995	chlorophyll biosynthetic process	biological_process	15	6	2.5
GO:0019219	regulation of nucleobase-containing compound metabolic process	biological_process	150	65	2.3
GO:0031323	regulation of cellular metabolic process	biological_process	166	71	2.3
GO:0031326	regulation of cellular biosynthetic process	biological_process	147	65	2.3
GO:0032502	developmental process	biological_process	41	17	2.4
GO:0032989	cellular component morphogenesis	biological_process	15	11	1.4
GO:0044767	single-organism developmental process	biological_process	41	17	2.4
GO:0048856	anatomical structure development	biological_process	28	14	2.0
GO:0048869	cellular developmental process	biological_process	23	13	1.8
GO:0050789	regulation of biological process	biological_process	363	149	2.4
GO:0050794	regulation of cellular process	biological_process	358	148	2.4

GO:0051171	regulation of nitrogen compound metabolic process	biological_process	155	66	2.4
GO:0051252	regulation of RNA metabolic process	biological_process	145	63	2.3
GO:0060255	regulation of macromolecule metabolic process	biological_process	161	67	2.4
GO:0065007	biological regulation	biological_process	388	156	2.5
GO:0080090	regulation of primary metabolic process	biological_process	160	67	2.4
GO:1903506	regulation of nucleic acid-templated transcription	biological_process	138	63	2.2
GO:2000112	regulation of cellular macromolecule biosynthetic process	biological_process	147	65	2.3
GO:2001141	regulation of RNA biosynthetic process	biological_process	138	63	2.2
GO:0005634	Nucleus	cellular_component	179	76	2.4
GO:0005667	transcription factor complex	cellular_component	89	45	2.0
GO:0010007	magnesium chelatase complex	cellular_component	13	6	2.2
GO:0043227	membrane-bounded organelle	cellular_component	256	115	2.2
GO:0043231	intracellular membrane-bounded organelle	cellular_component	256	115	2.2
GO:0043235	receptor complex	cellular_component	9	4	2.3
GO:0044434	chloroplast part	cellular_component	13	6	2.2
GO:0044435	plastid part	cellular_component	13	6	2.2
GO:0098802	plasma membrane receptor complex	cellular_component	9	4	2.3
GO:1902911	protein kinase complex	cellular_component	11	7	1.6
GO:0000988	transcription factor activity, protein binding	molecular_function	26	13	2.0
GO:0000989	transcription factor activity, transcription factor binding	molecular_function	22	12	1.8
GO:0001071	nucleic acid binding transcription factor activity	molecular_function	68	35	1.9
GO:0001883	purine nucleoside binding	molecular_function	170	74	2.3

GO:0003700	transcription factor activity, sequence-specific DNA binding	molecular_function	68	35	1.9
GO:0003712	transcription cofactor activity	molecular_function	22	11	2.0
GO:0008138	protein tyrosine/serine/threonine phosphatase activity	molecular_function	12	7	1.7
GO:0015144	carbohydrate transmembrane transporter activity	molecular_function	11	6	1.8
GO:0016773	phosphotransferase activity, alcohol group as acceptor	molecular_function	72	36	2.0
GO:0016851	magnesium chelatase activity	molecular_function	13	6	2.2
GO:0017076	purine nucleotide binding	molecular_function	175	76	2.3
GO:0032549	ribonucleoside binding	molecular_function	170	74	2.3
GO:0032550	purine ribonucleoside binding	molecular_function	170	74	2.3
GO:0032553	ribonucleotide binding	molecular_function	172	75	2.3
GO:0032555	purine ribonucleotide binding	molecular_function	170	74	2.3
GO:0035639	purine ribonucleoside triphosphate binding	molecular_function	170	74	2.3
GO:0043168	anion binding	molecular_function	203	85	2.4
GO:0043565	sequence-specific DNA binding	molecular_function	48	24	2.0
GO:0051002	ligase activity, forming nitrogen-metal bonds	molecular_function	13	6	2.2
GO:0051003	ligase activity, forming nitrogen-metal bonds, forming coordination complexes	molecular_function	13	6	2.2
GO:0051087	chaperone binding	molecular_function	5	5	1.0
GO:0051537	2 iron, 2 sulfur cluster binding	molecular_function	9	5	1.8
GO:0097367	carbohydrate derivative binding	molecular_function	184	82	2.2
GO:1901476	carbohydrate transporter activity	molecular_function	11	6	1.8

DEG: Differentially expressed gene; RL: Red line; YL: Yangxia line.
